# PERC rule to exclude the diagnosis of pulmonary embolism in emergency low-risk patients: study protocol for the PROPER randomized controlled study

**DOI:** 10.1186/s13063-015-1049-7

**Published:** 2015-11-25

**Authors:** Yonathan Freund, Alexandra Rousseau, France Guyot-Rousseau, Yann-Erick Claessens, Olivier Hugli, Olivier Sanchez, Tabassome Simon, Bruno Riou

**Affiliations:** Paris Sorbonne Université, UPMC univ-Paris 6, UMRS INSERM 1166, IHU ICAN, Paris, France; Emergency Department, Hôpital Pitié-Salpêtrière, Assistance Publique-Hôpitaux de Paris (APHP), Paris, France; Plateforme de recherche clinique de l’est parisien (URCEST-CRCEST), Hôpital St Antoine, APHP, Paris, France; Emergency Department, Princess Grace Hospital, Monte Carlo, Monaco; Emergency Department, Lausanne University Hospital, Lausanne, Switzerland; Pneumology and Intensive Care Unit, Hôpital Européen Georges Pompidou, APHP, Université Paris Descartes, Sorbonne Paris Cite, Paris, France

## Abstract

**Background:**

The diagnosis of Pulmonary Embolism (PE) in the emergency department (ED) is crucial. As emergency physicians fear missing this potential life-threatening condition, PE tends to be over-investigated, exposing patients to unnecessary risks and uncertain benefit in terms of outcome. The Pulmonary Embolism Rule-out Criteria (PERC) is an eight-item block of clinical criteria that can identify patients who can safely be discharged from the ED without further investigation for PE. The endorsement of this rule could markedly reduce the number of irradiative imaging studies, ED length of stay, and rate of adverse events resulting from both diagnostic and therapeutic interventions. Several retrospective and prospective studies have shown the safety and benefits of the PERC rule for PE diagnosis in low-risk patients, but the validity of this rule is still controversial. We hypothesize that in European patients with a low gestalt clinical probability and who are PERC-negative, PE can be safely ruled out and the patient discharged without further testing.

**Methods/Design:**

This is a controlled, cluster randomized trial, in 15 centers in France. Each center will be randomized for the sequence of intervention periods: a 6-month intervention period (PERC-based strategy) followed by a 6-month control period (usual care), or in reverse order, with 2 months of “wash-out” between the 2 periods. Adult patients presenting to the ED with a suspicion of PE and a low pre test probability estimated by clinical gestalt will be eligible. The primary outcome is the percentage of failure resulting from the diagnostic strategy, defined as diagnosed venous thromboembolic events at 3-month follow-up, among patients for whom PE has been initially ruled out.

**Discussion:**

The PERC rule has the potential to decrease the number of irradiative imaging studies in the ED, and is reported to be safe. However, no randomized study has ever validated the safety of PERC. Furthermore, some studies have challenged the safety of a PERC-based strategy to rule-out PE, especially in Europe where the prevalence of PE diagnosed in the ED is high. The PROPER study should provide high-quality evidence to settle this issue. If it confirms the safety of the PERC rule, physicians will be able to reduce the number of investigations, associated subsequent adverse events, costs, and ED length of stay for patients with a low clinical probability of PE.

**Trial registration:**

NCT02375919.

## Background

### Pulmonary embolism in the emergency department

The incidence of pulmonary embolism (PE) in France and Europe has been estimated to 0.6–0.9 per 1000 persons per year [1, 2]. PE is a potentially lethal diagnosis [3], and its diagnosis in the Emergency Department (ED) is challenging [4].

**Fig. 1:**
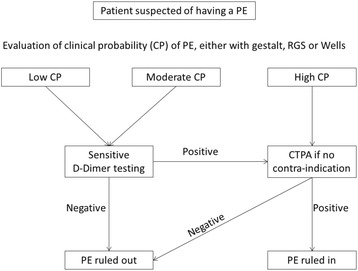
Standard strategy for the diagnosis of pulmonary embolism (PE) in the Emergency Department (ED). ED, Emergency Department; PE, pulmonary embolism; RGS, Revised Geneva Score

The fear of missing this diagnosis and the poor specificity of its clinical presentation has led physicians to suspect PE in patients who present with a broad variety of symptoms such as dyspnea, chest pain, syncope, and hypotension. These patients account for more than 10 millions ED visits a year in the United States. For the last decade, the strategy for PE diagnosis has been well-defined (Fig. 1). The usual work-up for PE diagnosis first includes an assessment of the clinical probability of PE, using a structured score (the Revised Geneva Score (RGS) or Wells’ score [5, 6]), or an unstructured estimation of the clinical probability (referred to as the clinician “gestalt” [7–9]). In patients with low clinical probability to moderate clinical probability, sensitive D-dimer testing should be followed if positive by a Computed Tomography Pulmonary Angiogram (CTPA) in the absence of contra-indication. Patients with a high clinical probability should undergo CTPA without the need for preliminary testing. This diagnostic strategy is recommended by European guidelines [10], national expert recommendations [11] and local policies. It has been validated and is safe to exclude PE in outpatients visiting the ED [12]. However, due to its low specificity (40–60 %) [9, 13], D-dimer testing may lead to more than 50 % of false positives and subsequent CTPA [9]. Furthermore, the wide availability of D-dimer testing, combined with the fear of missing PE, has led to lowering the testing threshold for suspicion of PE; hence the decrease in the prevalence of confirmed PE from 30 % to below 10 % in the United States [14–17].

Subsequently, there has been a marked rise (up to 15-fold) in the utilization of CTPA in the last 15 years [18] and in the incidence of diagnosed PE [19]. However, this greater incidence of PE was not followed by a decrease in the mortality rate from PE, but rather a global decrease in PE fatality [19, 20]: the prognosis of a patient with a PE improves, but the overall number of deaths from PE do not change. This suggests that PE tends to be “overdiagnosed”: small PEs are more frequently diagnosed, with no clear benefit in terms of outcomes. This increased exposure to CTPA may be a source of unnecessary risks, such as contrast-induced nephropathy and allergic reactions, adverse events after anticoagulation treatment or the delayed occurrence of radiation-induced cancer [21–23].

### The PERC rule

Assessing the benefit risk ratio for PE investigation, it has been calculated (using the Pausker and Kassirer method [24]) that if the pre test probability (PTP) is below 1.8 %, patients should not undergo D-dimer testing because a positive result would mandate a CTPA, which would have a negative benefit risk ratio [15]. To reduce the rate of unnecessary testings for PE caused by overuse of D-dimer, in 2004 Kline et al. developed a block rule of 8 binary variables (PERC rule): age < 50 years, pulse < 100 bpm, arterial oxygen saturation (SpO_2_) > 94 %, no unilateral leg swelling, no hemoptysis, no recent trauma or surgery, no prior PE or deep venous thrombosis (DVT) and no exogenous estrogen use [15] – PERC-negative patients are defined as fulfilling these 8 criteria. Kline et al. applied this rule in their princeps study to low-clinical-probability patients, defined by a probability of less than 15 % by empirical clinical gestalt [15, 16]. They reported that the prevalence of PE for PERC-negative patients was 1.4 % (95 % confidence interval (CI) 0.5–3 %). This low rate suggests that PERC-negative patients could be safely discharged after clinical examination without further testing. Moreover, the rate of 1.4 % is below the reported upper limit of false-negative rates after pulmonary angiogram or CTPA (3 %) [12, 25, 26], which advocates for a safe alternative to further testing.

Following this princeps study, several other studies assessed the safety of a PERC-based policy to exclude PE in low-risk emergency patients with a PERC-negative rule. Two meta-analyses [17, 27] confirmed the benefits and safety of the PERC rule, with a rate of PE after follow-up lower than 1 % in PERC-negative patients. They included 10 prospective and 3 retrospective studies, i.e. non-interventional studies only, accounting for a total of 14,844 ED patients with a suspicion of PE. Three of the 13 studies were conducted in Europe (France, Belgium and Switzerland) [28–30]. In the first 2 European studies [28, 29] the prevalence of PE amongst PERC-negative patients was 5.4 % and 6.7 %, respectively (95 % CI 3–10 %). Their authors argued that the higher prevalence of PE in Europe (>20 % [17]) than in the United States (<10 % [17]) was the main reason for this lower negative predictive value, and that this rule should not be applied in high-prevalence populations. Based on the poor performance of the PERC rule, European physicians have been reluctant to apply it for excluding PE in low-risk patients. However, these two studies had several methodological bias: both studies were retrospective and did not collect PERC items prospectively. Moreover, the studied samples were not solely patients with a low gestalt clinical probability: they included unselected patients with suspected PE in the ED, with low PTP to high PTP. Although authors from one study ran a sensitivity analysis focusing on patients with low PTP, this was assessed using the Revised Geneva Score (RGS) that is based on redundant items with those of the PERC rule. These specific limits, coupled with the greater prevalence in the European studies, might explain the greater rate of false negatives. A few years later, Penaloza et al. reported that the PERC rule was safe even in Europe, when combined with a low clinical probability assessed by physician’s gestalt [30], with no venous thromboembolic (VTE) event after 3-month follow-up. Accordingly, in a recent multicenter retrospective study, we also observed a very low prevalence of PE (0.5 % (95 % CI 0.1–1.1 %)) amongst low-risk, PERC-negative ED patients [31].

**Fig. 2:**
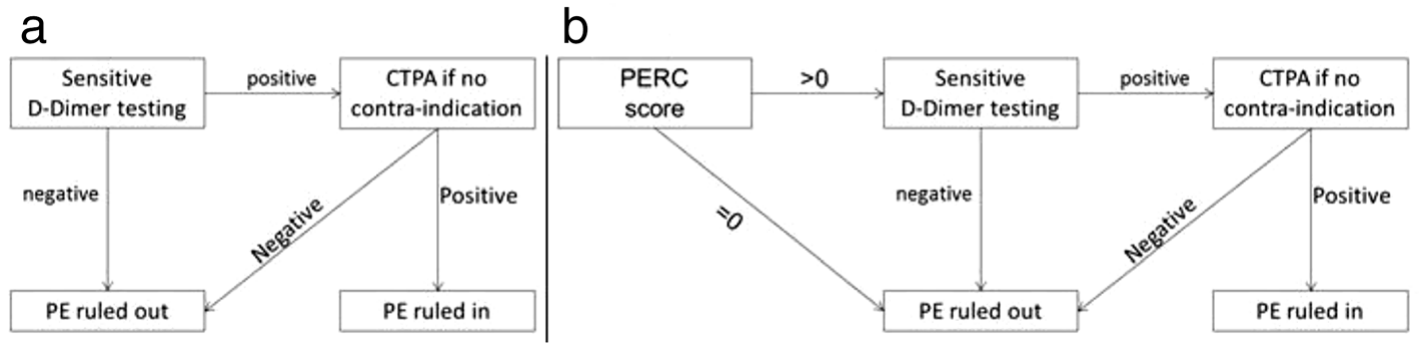
Work-up for diagnosis of pulmonary embolism (PE). **a** Control group. **b** Intervention group. CTPA, Computed Tomography Pulmonary Angiogram; PE, pulmonary embolism; PERC, Pulmonary Embolism Rule-out Criteria

Of note, all of the previously cited studies were either prospective or retrospective, but no randomized study has yet compared the benefit risk ratio of a PERC-based strategy versus the standard diagnostic strategy (Fig. 2) on occurrence of undiagnosed PE in low-risk patients.

## Methods

The PROPER trial is a cluster randomized trial in France. The primary objective of this study is to assess the non-inferiority of a PERC-based diagnostic strategy for PE low-risk emergency patients, compared to the standard strategy of D-dimer testing, on the occurrence of undiagnosed VTE events. Our institutional review board authorized the study with the need for a signed informed consent from the patient (Comité de protection des personnes, Paris Ile de France 6, A00215-44).

### Experimental plan

Each center will be randomized for the period: a 6-month intervention period followed by a 6-month control period, or a 6-month control period followed by a 6-month intervention period. The randomization will be prepared by URC-Est using permutation blocks (SAS 9.3, SAS Inc., Cary, NC, USA) before the first site initiation visit. The design, conduct and reporting of this study will follow the Consolidated Standards of Reporting Trials (CONSORT) statement extended to cluster randomized trials [32]. The two groups will have a different work-up for the diagnosis of PE in the ED as follows:

- Control group: standard strategy: conventional PE work-up. Every low-risk patient will undergo sensitive D-dimer testing, with subsequent CTPA if positive. In case of negative D-dimer, PE will be considered as excluded (Fig. 2a).
- Intervention group (PERC-based strategy): PE work-up based on the use of the PERC rule. If all PERC criteria are negative, no further testing for PE will be recommended. If at least one criterion is positive, then the patient will undergo sensitive D-dimer testing, with subsequent CTPA if positive. In case of negative D-dimer result, PE will be considered as excluded (Fig. 2b).

It has been recently reported that the cut-off for positive D-dimer should be changed for patients aged > 50 years to age × 10 ng/ml [33]. This strategy has been validated in a large multicenter international trial [34]. This strategy is actually endorsed by our centers. The strategy for defining “positive D-dimer” (age × 10 ng/ml for patients older than 50 years) will be stated before the start of the study in each center and not changed during the whole study period. Of note, this change will only concern patients aged 50 years or greater, i.e. with a PERC > 0. As this will not affect PERC-negative patients, there will be no interference with our objectives.

### Selection of participants

All patients with chest pain or dyspnea who attend one of the participating centers will be screened for eligibility by ED physicians and research assistants. If the treating ED physician or local investigator considers that the patient has a sufficient clinical suspicion of PE and that he needs a formal work-up for this diagnosis, and that this suspicion is low enough to discard this suspicion in case of negative D-dimer (i.e. estimated as less than 15 %), then the patient will be eligible. When a patient is eligible, his written informed consent will be obtained. In case of inability to consent, the patient will not be included in the study. The inclusion criteria are as follows:

- Patients ≥ 18 years presenting to an ED AND
- New onset of, or worsening of shortness of breath or chest pain AND
- A low clinical probability of PE, estimated by the clinician gestalt (unstructured empirical probability of PE) to be lower than 15 %

Exclusion criteria include the presence of any obvious cause other than a PE of symptoms, an acute severe presentation (clinical signs of respiratory distress, hypotension, SpO2 < 90 %, shock), a contraindication to CTPA (allergy, or estimated creatinine clearance less than 30 ml/min), pregnancy, concurrent anticoagulation treatment, current diagnosis of VTE event, inability to follow-up or to provide informed consent, lack of coverage for medical insurance, being a prisoner, and participation in another intervention trial.

After a patient is screened in the ED and may be included, the patient’s emergency physician, the local investigator or a clinical research technician (CRT) will inform them of the trial, and obtain signed informed consent. After a patient is included in the study, the local investigator, the patient’s emergency physicians, or the CRT will collect data on their past medical history, vital signs on admission, PERC, RGS, Wells’ score, examination in the ED and discharge disposition. This will be collected on a paper Case Report Form (CRF), later entered on an electronic CRF (eCRF). Any missing data will be sought through electronic or paper records of the patients, under the supervision of the local investigator of the center.

### Trial objectives and outcomes

The primary objective of this study is to assess the non-inferiority of a PERC-based diagnostic strategy for ED patients with a low probability of PE based on physician’s gestalt, compared to the standard strategy of D-dimer testing, on the prevalence of undiagnosed VTE events. The secondary objectives are the following:

1. to assess the reduction of unnecessary irradiative imaging studies and adverse events
2. to assess the reduction in ED length of stay
3. to assess the reduction of onset of anticoagulation regimen and associated adverse events
4. to assess the reduction of hospital admission following the ED visit, hospital readmission, and mortality at 3 months.

The primary outcome is the percentage of diagnostic strategy failure, defined as diagnosed VTE at 3-month follow-up, among patients for whom PE has been initially ruled-out. Exclusion of PE in the ED is made based upon a negative D-dimer result or a negative CTPA in both groups, or a negative PERC in the intervention group.

Follow-up will be made by telephone interview of the patient or his general practitioner, and review of any outpatient consultation or hospital visit at 3 months (13 weeks) by a CRT. The time frame of the 3-month follow-up period could be subject to minor adjustments, but should occur between day 84 and day 98. Follow-up visit or interview will seek the occurrence of VTE events (DVT documented with ultrasonography of the lower limbs or venous computed tomography (CT), or PE documented with positive CTPA or high probability ventilation/perfusion (V/Q) lung scan), death, return visit to the ED, and hospitalization. All medical records pertaining to the patient within this timeframe will be sought and analyzed by the local investigator, looking for reports of VTE events, or adverse events from CTPA or anticoagulation. In case of death, report of a VTE, or major cardiovascular event, the file will be analyzed by a committee of three independent experts. This method of adjudication has been described and validated in all major previous PE diagnostic studies [35, 36].

The primary criterion of a VTE event will be based on an objective diagnosis of DVT on Doppler ultrasonography, an intraluminal defect on CTPA, or a V/Q lung scan with a reported high probability. To confirm the occurrence of the primary endpoint, all files with evidence of a thromboembolic event collected by the local investigator of each center will be independently reviewed by an adjudication committee of three experts, blinded one to the other, and blinded to the study group. The adjudication committee will also review cases of death with no evidence of VTE event and will adjudicate whether or not the death is likely related to a PE. A sudden death in the absence of other obvious cause will be adjudicated as related to a PE. This committee will include three members, independent from the trial, with expertise in the field of PE, and will meet the recommendations of Dechartres et al. [37].

The result of adjudication regarding the primary endpoint, and other secondary endpoints at follow-up will be entered on the eCRF by a CRT or the local investigator.

### Statistical analysis

No interim analysis is planned.

Baseline characteristics of patients will be described according to group of intervention. Continuous variables will be summarized using descriptive statistics, i.e. number of subjects, mean, median, standard deviation (SD), interquartile range, minimum and maximum. Qualitative variables will be summarized by frequency and percentage. Since this is a non-inferiority study, analysis of the principal criterion will be performed on a per-protocol population. Secondary analysis will be performed based on the intention-to-treat (ITT) principle. VTE events will be defined by: DVT (assessed by proximal compression ultrasonography) or PE (a CTPA or angiography showing intraluminal defect, or a V/Q lung scan showing a high-probability pattern). The decision rule will be based on the upper bound of the 90 % 2-sided CI of the difference of percentage of VTE events between groups. If the upper bound of the CI is above the 1.5 % of difference, the non-inferiority hypothesis of the intervention group will be rejected. The Dunnett and Gent chi square test will also be performed. Secondary analysis will be performed on the ITT population. Considering cluster randomization, confirmatory analysis will be performed using a generalized estimating equation (GEE) assuming an exchangeable correlation matrix structure and considering clustering at the site level. Secondary criteria will be compared under superiority hypothesis and on the ITT population. Descriptive analysis will be performed. Superiority approach will be used to compare secondary evaluation criteria between groups. The ED length stay and the mean proportion of hospital admissions following the ED visit will be compared using a mixed model, considering “center” as random effect. Unnecessary irradiative imaging, adverse events and deaths at 3 months will be compared using GEE assuming an exchangeable correlation matrix structure and considering clustering at site level. All superiority tests will be performed at 5 %. Pre-specified secondary analysis will include comparison of gestalt, Wells’ score and RGS, and performances of the PERC35 score for patients aged under 35 years [38]. Missing data will not be replaced except for the principal criteria for the secondary ITT analysis. Missing value will be considered as an event whatever the group randomized.

According to recent large European cohorts, we estimate that the primary endpoint rate in our control group will be 1.5 % [35, 36, 39]. To be regarded as non-inferior, the maximal difference in proportions between 2 groups (Delta) should not exceed 1.5 % – an absolute primary event rate of 3 % in the intervention group. This failure rate corresponds to the upper bound of the observed rate after a negative CPTA and is a widely accepted criterion for the validation of diagnostic strategies for PE [40]. This rate is in line with previous landmark studies that comprise the basis of our current understanding.

### Sample size under non-inferiority hypothesis

To assess non-inferiority of the “PERC strategy,” with 1-sided alpha = 5 %, beta = 20 %, *N1* = 1624 subjects are needed (East 6, Cytel, Cambridge, MA, USA). A cluster is a 6 months period for 1 site. Under the assumption of an intraclass correlation coefficient of 0.002, an intra-period correlation of 0.001 and a mean cluster size for one period of 60 patients, the cluster design effect would be of *D* = 1.118. Considering 5 % of non-evaluable subjects, with 15 sites involved in the trial, which corresponds to 30 clusters, 61 subjects per site per period are required and will lead to a total of 1920 patients.

## Discussion

PE is a diagnosis that affects nearly 200,000 patients each year in France. The multiplication of diagnostic studies led to a rise in PE diagnosis, associated with a concurrent rise in the diagnosis of minor PE, and no subsequent decrease in mortality [18–20].

According to many retrospective studies including ours, the rate of PERC-negative patients amongst patients with a low clinical probability of PE ranges from 15–30 % [15, 28, 30, 31]. If the PERC rule was used in place of a conventional D-dimer-based diagnostic strategy, more than 10 % of CTPAs could be avoided [31, 41].

Such reduction in imaging studies would be beneficial for patients. The main medical harms that can be caused by unnecessary testing for PE include adverse events from CTPA: increased risk of delayed solid tumor occurrence from irradiative imaging, and iatrogenic complications of anticoagulation for positively tested patients (either false positives or true positives for small PEs). Moreover, the benefits of diagnosing PE in low-risk patients are unclear. Mortality for patients with suspicion of PE seems very low: Kline reported that among more than 8000 patients tested for PE in the ED, only 13 patients (0.2 %) died because of PE (*written communication*).

In 2011, Newman and Schriger extrapolated the risks and benefit of D-dimer testing among a sample of 10,000 PERC-negative patients [41]. This supplemental testing could lead to the diagnosis of 30 PE that would have been missed (credible interval 6–60). However, further testing in these 10,000 PERC-negative patients may cause 73 adverse events (credible interval 14–140) among which 36 fatal events (credible interval 4–69). The causes of adverse events were acute renal failure from contrast-induced nephropathy (50 per 10,000 patients), severe hemorrhage due to anticoagulation treatment (17 per 10,000 patients), and cancer resulting from radiation (5 per 10,000 patients).

Besides the estimated unfavorable medical benefit risk ratio for the patient, further testing has clear downsides: a prolonged stay in the ED, contributing to overcrowding [42, 43], overall worse short-term outcomes [44], and increased costs. In a retrospective study, median CTPA time in the ED has been reported to be 160 minutes, accounting for more than half of total ED [41]. ED length of stay could be greatly reduced if PERC was endorsed: nearly a quarter of patients with a low PTP could be discharged after just a physical examination, without the need for time-consuming biological and imaging studies.

Finally, avoiding any supplemental investigations for PERC-negative patients may also reduce the costs of ED visits, which would be of great benefit in the context of increasingly resource-stretched healthcare services. Thus, if the PERC-based strategy is shown to be non-inferior to the standard strategy, it will safely and substantially reduce the volume of D-dimer and CTPA testing and, therefore, irradiation, adverse events, length of ED stay and overcrowding.

The risk for a patient recruited in the experimental group is that of a false negative PERC score, whilst the patient actually has a PE. This risk has been reported to be below 1 % in previously cited meta-analyses. Furthermore, potential false-negative patients would belong to the group at lower PE risk, with an estimated 30 days mortality below 1 % [45–47] – on top of the overall mortality rate estimated in the conventional group (0.2 %), the overall extrapolated added risk would be below 1/10,000 at 30 days in the experimental group.

The PROPER study is a large international cluster randomized controlled trial that aims to validate the safety of the PERC rule to exclude PE in emergency patients with a low clinical probability. This trial is the first to prospectively implement and evaluate PERC in a controlled fashion to validate what previous meta-analyses have reported, and may end the controversy in this European high-prevalence population on its safety.

## Trial status

Recruiting.

## Abbreviations

CONSORT: Consolidated Standards of Reporting Trials
CRF: Case Report Form
CRT: clinical research technician
CT: computed tomography
CTPA: Computed Tomography Pulmonary Angiogram
DVT: deep venous thrombosis
eCRF: electronic CRF
ED: Emergency Department
GEE: generalized estimated equation
ITT: intention-to-treat
PE: pulmonary embolism
PERC: Pulmonary Embolism Rule-out Criteria
PTP: pre test probability
RGS: Revised Geneva Score
SD: standard deviation
SpO_2_: arterial oxygen saturation
V/Q: ventilation/perfusion
VTE: venous thromboembolic
